# Electronic Structure and External Electric Field Modulation of Polyethylene/Graphene Interface

**DOI:** 10.3390/polym14142949

**Published:** 2022-07-21

**Authors:** Hongfei Li, Zhaoming Qu, Yazhou Chen, Linsen Zhou, Yan Wang

**Affiliations:** 1 National Key Laboratory on Electromagnetic Environment Effects, Army Engineering University, Shijiazhuang 050003, China; hongfeili0704@126.com (H.L.); iamqzm3990@163.com (Z.Q.); wyan8628@163.com (Y.W.); 2 Institute of Materials, China Academy of Engineering Physics, Mianyang 621907, China; zhoulinsen173@aliyun.com

**Keywords:** polymer nanocomposite, interface, field-shielding material, electric field modulation, carrier transport, first principle

## Abstract

Polymer nanocomposites can serve as promising electrostatic shielding materials; however, the underlying physical mechanisms governing the carrier transport properties between nanofillers and polymers remain unclear. Herein, the structural and electronic properties of two polyethylene/graphene (PE/G) interfaces, i.e., type-H and type-A, have been systematically investigated under different electric fields using first principle calculations. The results testify that the bandgaps of 128.6 and 67.8 meV are opened at the Dirac point for type-H and type-A PE/G interfaces, respectively, accompanied by an electron-rich area around the graphene layer, and a hole-rich area around the PE layer. Moreover, the Fermi level shifts towards the valence band maximum (VBM) of the PE layer, forming a p-type Schottky contact at the interface. Upon application of an electric field perpendicular to the PE/G interface, the Schottky contact can be transformed into an Ohmic contact via the tuning of the Schottky barrier height (SBH) of the PE/G interface. Compared with the A-type PE/G interfaces, the H-type requires a lower electric field to induce an Ohmic contact. All these results can provide deeper insights into the conduction mechanism of graphene-based polymer composites as field-shielding materials.

## 1. Introduction

In recent years, the rapid development of electronic devices making use of low-frequency microwave radiation—such as cellular phones, local area networks, and radar systems—has led to serious electromagnetic interference problems. Therefore, the interaction between polymer dielectric composites and microwaves has attracted considerable attention [1,2,3,4]. Composites of carbon-based nanoparticles and polymers have been extensively investigated as antistatic or electromagnetic shielding materials, owing to their excellent mechanical and electrical properties [5,6,7,8,9,10]. The combination of polymers and nanoscale inorganics leads to a “1 + 1 > 2” composite effect, with excellent dielectric properties. Despite extensive experimental studies [11,12,13,14,15,16], the mechanism of carrier transport between nanofillers and polymers is not yet completely understood.

The first principle simulation based on density functional theory (DFT) is a general method for determining the structures and interactions of materials [17,18,19,20,21], and has become a powerful tool for exploring their properties. Sun et al. [22,23] successfully established a correlation between the breakdown strength and band gap of selected materials based on first principles. Many first principle studies were conducted to explore the charge-transport properties of organic–inorganic composite systems, such as polyethylene/nanofiller interfaces [24,25,26], in search of the intrinsic relationship between the electrical breakdown and charge transport in specific materials. The charge trapping theory was then proposed by Kubyshkina et al. [27] to research polyethylene/MgO nanocomposites, where a long-range potential well up to 2.6 eV deep exists in the interfacial region. Sato [28] also studied the electronic structures of a polyethylene/MgO configuration to understand the charge distributions during the carrier doping and band arrangement at the interface.

Graphene can be embedded in polymers to enhance the electrical conductivity of composites owing to its own excellent conductivity, high carrier mobility, and high thermal conductivity. Its conduction and electric field break easily, which occurs at the graphene edge. Gaska [29] experimentally established that 1 wt% graphene nanosheets enhance the mechanical properties of low-density polyethylene (LDPE)-based composites and produced nonlinear field-dependent conductivity behavior. This strong-field-induced high-conductivity characteristic behavior could be applied to a smart shielding effect in complex electromagnetic environments. The interfacial region between the polymer matrix and inorganic nanofiller may play a key role in tuning dielectric properties [30,31]. However, the transport mechanisms at the interface between the organic layers and graphene continues to be a challenging issue.

To date, polyethylene (PE) and graphene (G) are used as one of the most widely studied polymeric dielectric materials. It is of crucial importance to understand their conduction states, since electrons are usually the relevant carriers for transport and breakdown behaviors. In this study, two types of polyethylene/graphene (PE/G) hybridization models, i.e., type-A and type-H, were constructed. The effects of the electric field on the electronic properties of the PE/G interface have been investigated using first principles calculations. The calculated results demonstrate that the contact type (Schottky/Ohmic) and barrier height of the PE/G interface could be effectively modulated by applying an external electric field. The purpose of this study on field-adjusted band alignment is to understand the nature of the conductive state of graphene-based polymer composites. Although the system is ideal to some extent, our results can provide deeper insights into the conduction mechanism of graphene-based polymer composites as field shielding materials. These studies may also stimulate further theoretical studies on these fields.

## 2. Materials and Methods

In this study, geometric and electronic calculations based on the density functional theory (DFT) were implemented in VASP code [32,33]. The exchange and correlation effects were treated using the Perdew–Burke–Ernzerhof (PBE) functional of the generalized gradient approximation (GGA) [34]. The Brillouin zones (BZs) of the type-H and type-A PE/G interfaces were sampled at 3 × 5 × 1 and 3 × 3 × 1 k-points, respectively. The vacuum space was set at 20 Å to minimize the slab-to-slab interaction. The van der Waals (vdW) correction factor was considered at the vdW-DF level [35,36]. All geometries were completely relaxed until the total energy converged to 10^−5^ eV and the maximum force was 0.001 eV/Å. The calculated lattice constants of graphene were *a*_G_ = 4.26 Å and *b*_G_ = 2.46 Å, respectively. The crystal structure parameters of polyethylene were *a*_PE_ = 7.18 Å, *b*_PE_ = 4.94 Å, and *c*_PE_ = 2.56 Å, respectively, which were in good agreement with previous results [37,38,39], listed in Table 1.

## 3. Results and Discussion

### 3.1. Structural Features of the PE/G Interfaces

**Figure 1:**
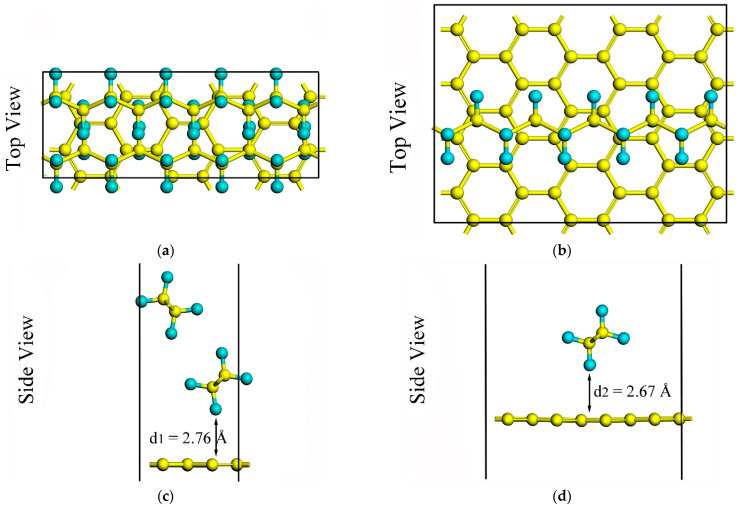
Top (upper panel) and side views (lower panel) of atomic geometries for the PE/G interfaces: (**a**,**c**) for type-H; (**b**,**d**) for type-A. Yellow and blue spheres represent C atoms and H atoms, respectively.

The optimized geometries of the PE/G interfaces are shown in Figure 1. Two hybridization models of graphene/polyethylene were constructed, one being a two-dimensional heterostructure formed by lattice matching of polyethylene and graphene (heterostructure type-H), and the other being a polyethylene chain adsorbed onto the graphene surface to form a complex (adsorption type-A). As shown in Figure 1a,c, a 3 × 2 graphene (24 carbon atoms) supercell was obtained to match a 5 × 2 PE supercell (2 chains composed of 20 −CH_2_− monomers) with a negligible lattice mismatch of <2%. The PE/G interface was constructed in an orthogonal supercell, and the lattice constants of that were *a* = 12.78 Å and *b* = 4.93 Å. For the type-A hybrid, the lattice constant of the 3 × 4 graphene supercell was *a* = 12.78 Å and *b* = 9.86 Å, and the adsorbed polyethylene chain exhibits a zigzag configuration consisting of 10 −CH_2_− monomers, which was plotted in Figure 1b,d. The corresponding key parameters of the type-H and type-A PE/G interfaces are listed in Table 2.

To effectively evaluate the interface mechanical properties, the binding energy (*E*_b_) can be estimated using the following formula:

(1) $$E_{b}{=E}_{\mathrm{PE}/G}-E_{\mathrm{PE}}-E_{G}$$

where *E*_PE/G_ correspond to the total energy of the PE/G interfaces, and *E*_PE_, and *E*_G_ are the energies of individual PE and G, respectively.

**Figure 2:**
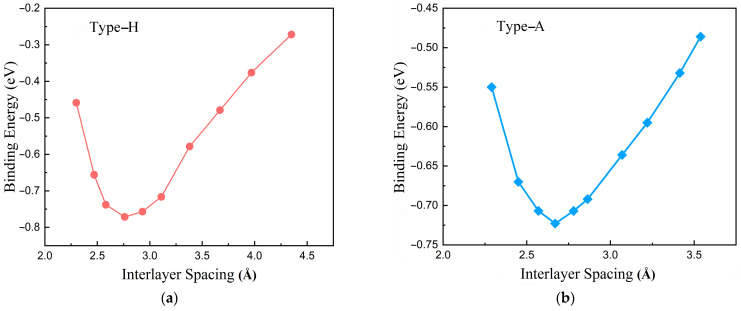
Binding energy (*E*_b_) as a function of the interlayer spacing between graphene and PE for (**a**) Type-H and (**b**) Type-A.

In the following procedure, the spacing distance between the graphene and PE was adjusted to detect the true equilibrium configuration. The binding energy as a function of layer spacing is plotted in Figure 2. Clearly, in the case of PE/G interfaces, the equilibrium interlayer distances for type-H and type-A are, respectively, *d*_1_ = 2.76 and *d*_2_ = 2.67 Å as listed in Table 1. Corresponding to the lowest binding energies of the two stacking modes are −0.76 and −0.72 eV, respectively. The most negative binding energy exhibits the best electronic stability of the favorable complexation of PE/G interfaces.

### 3.2. Electronic Structures of the PE/G Interfaces

**Figure 3:**
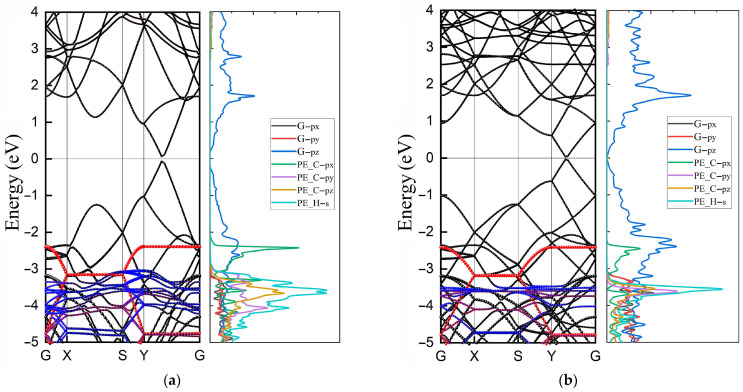
Band structures and PDOSs of (**a**) type-H and (**b**) type-A PE/G interfaces, respectively. The black curves denote graphene, while the red and blue solid dots denote the contribution from C and H atoms and states of PE in the band structures of PE/G interfaces.

Local perturbation of the electronic structure at the interfaces may significantly affect the electronic properties of composite materials. To obtain the electronic properties of the PE/G interfaces, the band structures of type-H and type-A PE/G interfaces were studied, as shown in Figure 3a,b, respectively. The Fermi level (*E*_f_) is set at 0 eV. The path along the high-symmetry points of the BZ is selected as Gamma (G)-X-S-Y-Gamma (G). A linear Dirac-like dispersion can be folded onto the Y-G path, which is confirmed by the band structure of other rectangular-lattice graphene [40]. Furthermore, the bandgaps of about 128.6 meV and 67.8 meV were opened at the Dirac point for the type-H and type-A PE/G interfaces, respectively, which may be induced by the reduced in-plane symmetry at the PE/G interface [41]. It is observed that the electronic states at the conduction band minimum (CBM) and valence band maximum (VBM) are mainly dominated by graphene. The PE is mainly located in the valence band, exhibiting a natural type-I band arrangement. The valence band edge of PE is at the energy of 2.4 eV, which mainly comes from the contribution of the 2p_x_ orbital of the C atom of PE, forming a p-type Schottky barrier height (SBH) at the PE/G interface. Notably, the presence of distinct flat bands in the PE located in the valence band, indicating the existence of considerable numbers of quantum states in PE with similar kinetic energies. This strong electron localization corresponds to the formation of spikes in the density of states. From the projected density of states (PDOS) of two-type PE/G interfaces, it is noted that the orbital overlap of PE and graphene is mainly located in the valence band. Therefore, understanding the regulation of the electronic state of the valence band is essential for the design of polymeric dielectric materials.

**Figure 4:**
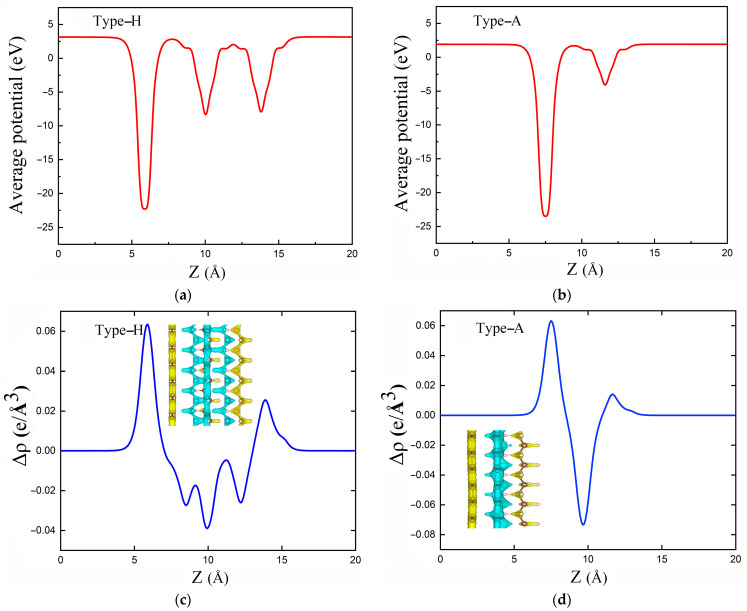
(**a**,**b**) the electronic potential energy distribution along the Z-direction, with the plane-averaged charge density in (**c**,**d**) for type-H and type-A PE/G interfaces, respectively. The inset is the charge density difference with an isosurface of 0.15 e/Å. The yellow and cyan areas represent electron accumulation and depletion, respectively.

The electrostatic potentials along the Z-direction perpendicular to the interface for the PE/G interfaces are shown in Figure 4a,b. The results show that large potential drops of 14.0 and 19.4 eV are formed at the type-H and type-A interfaces, respectively, indicating the presence of a strong electrostatic field at the interface. The carrier dynamics at the interface are significantly affected, leading to a redistribution of the charge density and the formation of interfacial dipoles in PE/G interfaces. The potential of graphene is lower than that of PE, so it is easier to transfer electrons from the PE to the graphene layer. In addition, the charge redistribution between the PE/G interfaces can be visualized by using the plane-averaged charge density, as shown in Figure 4c,d, which is calculated as

(2) $$\Delta {\rho =\rho }_{I}-\rho_{G}-{n\rho }_{\mathrm{PE}}$$

where the *ρ*_I_, *ρ*_G_, and *ρ*_PE_ are the charge density of the PE/G interfaces, monolayer graphene, and PE, respectively. The results show that the charges are mainly accumulated in the graphene layer and depleted in the PE layer. Ultimately, the graphene and PE layers form an electron-rich region and a hole-rich region, respectively. The electrons are transferred from the PE to the graphene layer at the PE/G interface, consistent with results of the electrostatic potential.

### 3.3. Electric Field Effects on the PE/G Interfaces

**Figure 5:**
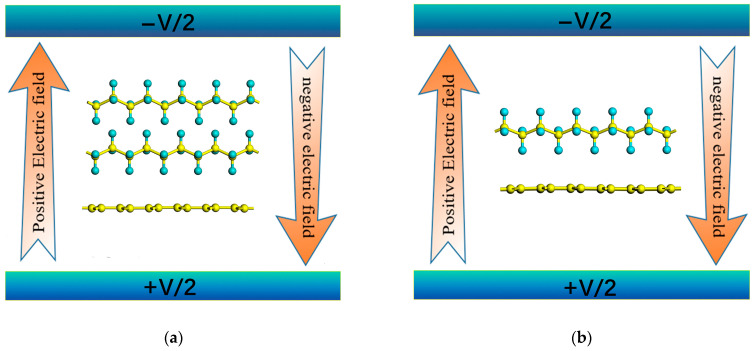
The schematic illustration of band structures of (**a**) type-H and (**b**) type-A PE/G interfaces under perpendicular electric field, respectively.

Now, we turn to investigate the modulation effect of the external electric field on the electronic structure of the PE/G interface. In Figure 5, we illustrate the schematic illustration of the external electric field to PE/G interfaces. The direction from the graphene to the PE layer was defined as the positive direction of the electric field, and vice versa.

**Figure 6:**
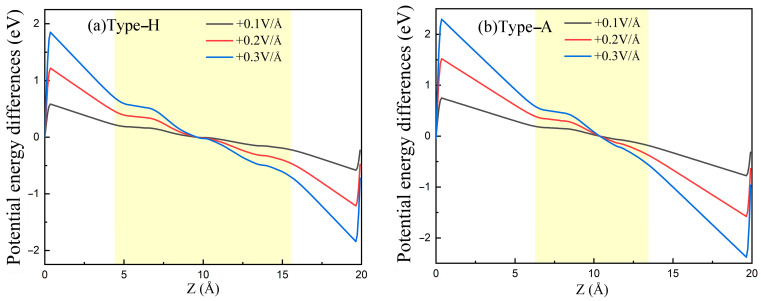

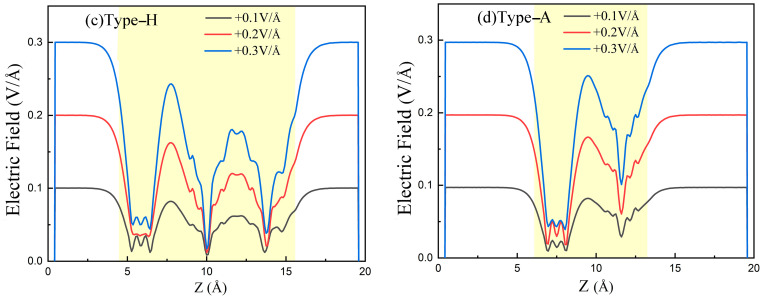
Upper panels (**a**,**b**) potential energy differences induced by various electric fields and lower panels (**c**,**d**) electric field intensity distribution in the Z-direction for type-H and type-A PE/G interfaces, respectively.

Firstly, we calculated the potential energy difference (∆*ε* = *ε*_E_ − *ε*_0_) along the Z-direction, as shown in Figure 6a,b. Here, *ε*_E_ and *ε*_0_ represent the electrostatic potential energy under electric fields of E V/Å and 0 V/Å, respectively. Further, the distribution of the electric field intensity along the Z-direction can be determined by differentiating the potential energy differences using equation:

(3) $$E=\frac{d\varphi }{dz}=\frac{d\Delta \varepsilon }{-edz}$$

In Figure 6, the regions corresponding to the PE/G interfaces’ distribution are highlighted in yellow. One can observe that the electric field intensity in a vacuum is practically equal to the applied electric field intensity, while that in the PE/G distribution region is obviously weakened. Then, the average electric field intensity in the yellow region are considered to be the effective electric field intensity. The calculations show that effective field strengths of the type-H and type-A regions were about 1/2.4 and 1/2 of the applied values, respectively. The reduction of the electric field in the PE/G interfaces can be attributed to the response field at the interface opposite to the external electric field, intuitively reflecting the electromagnetic shielding properties of the material.

**Figure 7:**
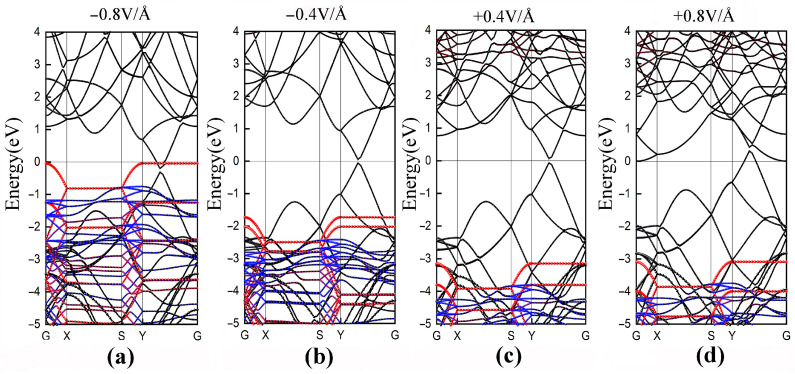
Band structures for type-H PE/G interfaces under electric fields of (**a**) −0.8 V/Å, (**b**) −0.4 V/Å, (**c**) +0.4 V/Å and (**d**) +0.8 V/Å.

It is well known that conventional DFT calculations usually underestimate the band gap [42], but here we only focus on the variation trends in the band structures. The band structures of the type-H PE/G interfaces under different applied electric fields are shown in Figure 7a–d. The results clearly show that with the increase of the negative electric field, more electrons overcome SBH (2.4 eV) and transfer the electrons from the valence band of PE to the Dirac point. Subsequently, the p-type SBH decreases and the VBM of PE approaches the Fermi level. The application of a −0.8 V/Å electric field can induce a transition from the Schottky contacts to an Ohmic contact. Opposite to the effect of the negative electric field, the electrons can be transferred from the Dirac point to the conduction band of PE. The conduction bands with graphene’s contribution show a clear shift down to the Fermi level, yet the VBM of PE shifts downward a little bit. Interestingly, as shown in Figure 7a,d, the Dirac cones of graphene are located at different positions under opposite electric field directions, higher than the Fermi level which exhibits semi-metallic character and lower than the Fermi level which proves the metallic character. In fact, this is consistent with previous reports [43,44]. External electric fields induce charge transfer in interface and the electrostatic potential felt by electrons changes accordingly, resulting in energy-level shifting of CBM and VBM. The evolution of potential barrier heights can be more intuitively associated with the breakdown performance of the dielectric materials. 

**Figure 8:**
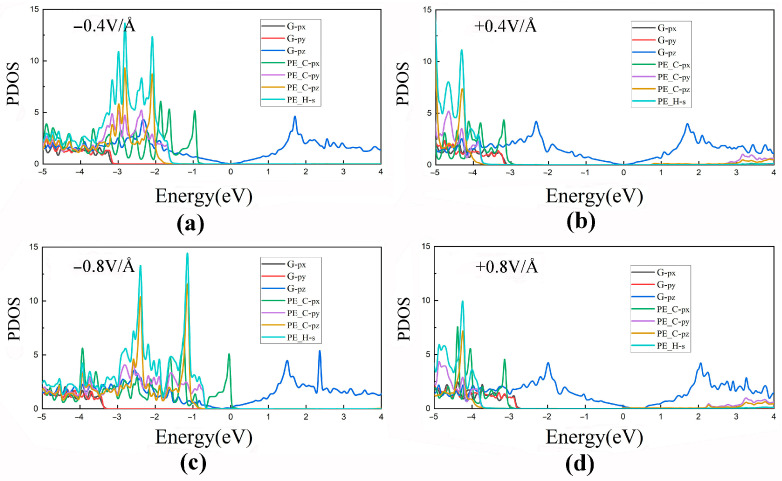
PDOSs for type-H PE/G interfaces under electric fields of (**a**) −0.4 V/Å, (**b**) +0.4 V/Å, (**c**) −0.8 V/Å and (**d**) +0.8 V/Å.

To better describe the effect of the external electric field on electronic properties of PE/G interfaces, the PDOSs of the type-H PE/G interfaces under applied electric field were further studied, as presented in Figure 8a–d. When the PE/G interface is subjected to the negative electric field, the orbitals of both the C and H atoms of PE shift towards the Fermi level, in which the 2p_x_ orbital of the C atom dominates and crosses the Fermi level under the −0.8 V/Å electric field, so that the p-type contact changes into an Ohmic contact. By contrast, the 2p_y_ and 2p_z_ orbitals of C atoms of PE move towards the Fermi level in the conduction band with an increasing positive electric field. On the other hand, the PE orbitals in the valence band region see little change, while the 2p_y_ orbital of graphene shift closer to the Fermi level. The results are in good agreement with the band structures of type-H PE/G interfaces.

**Figure 9:**
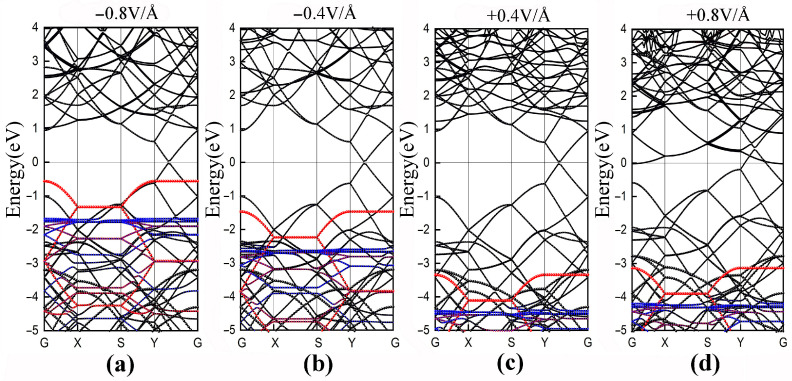
Band structures for type-A PE/G interfaces under electric fields of (**a**) −0.8 V/Å, (**b**) −0.4 V/Å, (**c**) +0.4 V/Å and (**d**) +0.8 V/Å.

**Figure 10:**
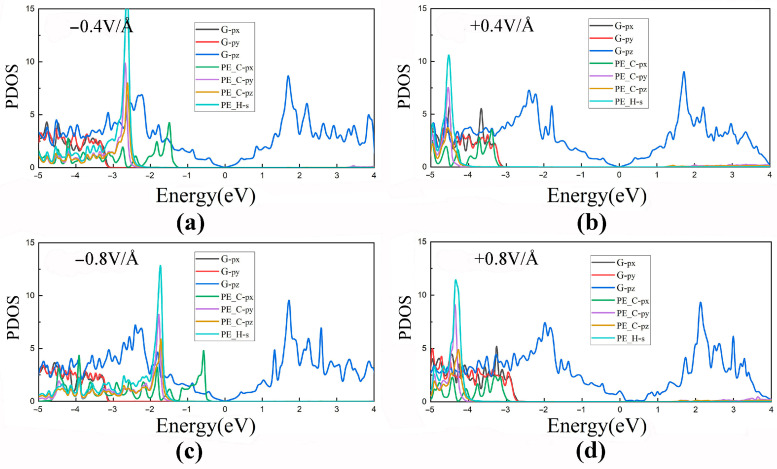
PDOSs for type-A PE/G interfaces under electric fields of (**a**) −0.4 V/Å, (**b**) +0.4 V/Å, (**c**) −0.8 V/Å and (**d**) +0.8 V/Å.

In this section, the electronic structures of the type-A PE/G interface were examined under different electric fields, as shown in Figure 9a–d. It is obvious that the VBM of PE gradually rises near the Fermi level with negative electric fields, while with a positive electric field the CBM of graphene plays a dominant role and decreases near the Fermi level. The PDOSs of the type-A PE/G interface are depicted in Figure 10a–d. The C-2p_x_ orbital of PE is close to the Fermi level under negative electric field, by contrast, the 2p_x_ and 2p_y_ orbitals of graphene approach the Fermi level by applying positive electric field. When the applied electric field is +0.8 V/Å, the 2p_z_ orbitals of graphene crosses the Fermi level to induce half-metallicity. From the above discussion, we know that the SBH can be reduced in the negative electric field, and it is easier to induce an Ohmic contact. Compared with type-H PE/G interfaces, type-A requires a higher electric field to switch the Schottky barrier to form an Ohmic contact.

## 4. Conclusions

The electronic properties of graphene/polyethylene interfaces under external electric fields have been examined using DFT calculations. Two hybridization configurations, viz. type-H and type-A PE/G interfaces, were constructed. The lowest binding energies of −0.76 and −0.72 eV for type-H and type-A, respectively, signifies that considered PE/G interfaces can maintain electronic stability well. In addition, the stack of PE opens a bandgap of 128 and 67.8 meV at the Dirac point for the type-H and type-A PE/G interface, respectively. The Fermi level shifts towards the VBM of the PE layer, i.e., the Schottky contact is p-type with a large SBH (2.4 eV). The charge transfer forms an interfacial dipole, where the charge accumulated in the graphene layer and depleted in the PE layer. It is found that the charge polarization is sensitive to the strength and direction of the electric fields, and the negative electric fields can effectively modulate the SBH at the interface to achieve the Schottky-to-Ohmic contact transition, in which the 2p_x_ orbitals of the C atoms of PE play a dominant role. Compared with the type-A, the type-H interface surmounts the lower SBH to form an Ohmic contact. These studies provide important implications for understanding the field breakdown behaviors of graphene-based polymer composites.

## Figures and Tables

**Table 1:** Values of equilibrium lattice parameters *a*_0_, *b*_0_ and *c*_0_ of graphene and polyethylene crystal structure compared with other theoretical data.

	Graphene		Polyethylene		
*a*_0_ /Å	4.26 ^1^	4.26	7.12 ^2^	7.40 ^3^	7.18
*b*_0_/Å	2.46 ^1^	2.46	4.85 ^2^	4.93 ^3^	4.94
*c*_0_/Å	-	-	2.57 ^2^	2.53 ^3^	2.56

^1^ Ref. [37]. ^2^ Ref. [38]. ^3^ Ref. [39].

**Table 2:** The calculated lattice parameters (*a*, *b*), bond lengths of PE (*d*_C-C_, *d*_C-H_), interlayer distances (*d*), and binding energy (*E*_b_) of type-H and type-A PE/G interfaces.

	Type-H	Type-A
*a*/Å	12.8	12.8
*b*/Å	4.93	9.86
*d*_C-C_/Å	1.536	1.536
*d*_C-H_/Å	1.103	1.102
*d*/Å	2.76	2.67
*E*_b_/eV	−0.76	−0.72
